# Experimental Investigation of Puncture Behavior and Fractured Morphology of Steel/Graphene-Reinforced Polymer/Steel Sandwich Composite Structure

**DOI:** 10.3390/polym18182280

**Published:** 2026-09-18

**Authors:** Vu Hoai Anh, Nguyen Thuy Duong, Vu Toan Thang

**Affiliations:** 1 Precision Engineering & Smart Measurements Lab, School of Mechanical Engineering, Hanoi University of Science and Technology, Hanoi 100000, Vietnam; anh.vh240051d@sis.hust.edu.vn or hoaianh@uneti.edu.vn (V.H.A.); duong.nguyenthuy@hust.edu.vn (N.T.D.); 2 Faculty of Mechanical Engineering, University of Economics—Technology for Industries, Hanoi 100000, Vietnam

**Keywords:** sandwich composite structure, graphene-reinforced polymer, puncture behavior, fracture morphology, small punch test

## Abstract

Graphene has emerged as a revolutionary two-dimensional (2D) nanomaterial, profoundly altering the fields of materials science and mechanical engineering. Driven by its superior mechanical, electrical, and thermal properties, including a Young’s modulus of approximately 1 TPa, high electrical conductivity, and high thermal conductivity, researchers have extensively explored its potential as a reinforcing filler in various composites. The thin composite steel/graphene-reinforced polymer/steel sandwich structure proposed in this study can be applied in the development of precision electromechanical devices due to its advantages of high dimensional stability, high rigidity in a confined space, light weight, and ability to reduce micro-vibrations. This study evaluates the effect of adding graphene to the alkyd-based polymer core layer on the mechanical properties and fracture mechanisms of a sandwich system with a total measured thickness of 0.35 ± 0.01 mm. Through small punch tests (SPTs) with controlled die and punch geometry coefficients, the local load-bearing behavior of the material was investigated in detail. Experimental results show that the dispersion of graphene enhances the flexural stiffness of the core layer, thereby improving the flexural stiffness of the entire structure and increasing the maximum load by 12.6% (from 0.79 ± 0.03 kN to 0.89 ± 0.04 kN). However, this structure exhibits a clear mechanical trade-off as the fracture strain decreases from 1.80 ± 0.08 mm to 1.50 ± 0.09 mm, resulting in a slight 6.1% decrease in approximated energy absorption capacity. Morphological observations at the fracture groove suggest a shift in the fracture mechanism from macroscopic ductile tearing with large plastic deformation to localized abrupt brittle shear plug, accompanied by instantaneous elastic energy release and delamination. These findings indicate that the graphene-reinforced sandwich structure is suitable for thin-film applications requiring high static rigidity, but careful consideration is needed in environments subject to dynamic impact.

## 1. Introduction

In recent decades, materials science has developed dramatically from traditional materials to high-performance composites and nanocomposites [1,2]. Composites and nanocomposites are fundamental to advancing high-performance engineering applications, providing customized properties that monolithic materials cannot achieve. Composites, formed from the combination of two or more components with different properties, allow for the creation of superior properties that individual component materials cannot obtain [3,4]. The advent of nanocomposites, which have at least one reinforcing phase with a size at the nanometer scale (below 100 nm), has opened up the possibility of optimizing materials at the molecular level, meeting the stringent requirements in the aerospace, automotive, and electronics industries [5,6,7].

Although carbon nanotubes (CNTs) initiated the era of nano-reinforcement, their one-dimensional (1D) shape fundamentally limits their effectiveness. CNTs are severely entangled due to extremely strong van der Waals (VdW) interactions and provide a limited interface area for stress transmission.

The emergence of graphene in 2004 marked a great turning point in this field [2]. Defined as a single layer of carbon atoms arranged in a hybridized hexagonal lattice, graphene is considered the most ideal reinforcing material ever discovered [1]. Mechanically, it possesses a record Young’s modulus of approximately 1 TPa and an intrinsic tensile strength of up to 130 GPa [2,8]. In terms of graphene’s 2D planar structure, its enormous theoretical surface area (2630 m^2^/g) creates a huge interface region with the substrate material, which is key to achieving maximum load transfer efficiency [1,3].

When compared to traditional fillers (such as glass fibers and carbon fibers) or even carbon nanotubes (CNTs), graphene exhibits superior advantages [1,8]. While CNTs are 1D materials prone to entanglement and curling, graphene’s 2D flat sheet structure allows it to cover a larger surface area and create a tighter “locking” mechanical bond with the polymer or metal matrix; the 2D shape itself more effectively prevents crack propagation by forcing the cracks to zigzag (crack deflection and bridging mechanism) [4,9,10]. However, translating this theoretical potential into practical macrostructures continues to confront three primary engineering challenges: agglomeration induced by VdW forces, which causes graphene sheets to readily re-stack into graphite; the difficulty in achieving a homogeneous dispersion to produce a uniform mixture; and the chemical inertness of graphene, which reduces the efficiency of interfacial bonding for stress transfer from the matrix to the filler [1,3]. Despite these hurdles, by incorporating a small quantity of graphene into various host matrices (polymers, metals, ceramics), researchers can significantly enhance the properties of the base materials cost-effectively.

Based on these scientific foundations, this paper focuses on solving the problem of applying hybrid material structures by combining a graphene-reinforced polymer matrix with a thin steel shell. This study aims to clarify the changes in mechanical properties, particularly to evaluate the transformation in the fracture mechanism of ultrathin steel/polymer–graphene sandwich structures under the action of small punch loading.

Sandwich composite structures, especially systems using metal shells combined with a polymer core, play an important role in the development of ultralightweight structures that require high rigidity. Typical applications of these ultrathin sandwich structures include protective aerospace enclosures, lightweight automotive body panels, precision electronics casings, micro-electromechanical systems (MEMSs), and structural engineering applications for buildings, where high flexural rigidity-to-weight ratios are strictly required [11]. In these systems, the polymer core layer (typically alkyd or epoxy resin) plays a role in maintaining geometric stability and transmitting shear stress between the metal surfaces. However, original polymer matrices often have inherent limitations in stiffness and fracture toughness, leading to the risk of delamination or premature shear failure at the interface under mechanical load [12,13].

To overcome these drawbacks, carbon nanomaterials, particularly graphene and its derivatives, have emerged as ideal reinforcing fillers due to their superior mechanical, thermal, and electrical properties. Dispersing graphene sheets into polymer matrices has been shown to be an effective solution to significantly improve the elastic modulus, tensile strength, and microcracking resistance of composite materials. Graphene acts in polymer matrices as crack-bridging networks, thereby increasing the fatigue life and fracture toughness of the entire structure [14,15,16,17,18,19].

Besides the enhancement of the static mechanical properties of the core layer itself, the performance of the sandwich structure depends heavily on the interaction and stress transfer at the interfacial surface area. Shear-lag models and micromechanics have shown that the addition of graphene greatly increases the effective elastic modulus of the composite system. This explains why, under local puncture or bending loads, the graphene-stabilized core layer can resist compressive deformation better, allowing the structure to withstand higher critical load levels than conventional polymer cores [20,21,22].

Although the mechanical properties of graphene-reinforced polymer-based composites and graphene-reinforced metal-based composites have been extensively studied independently, the mechanical behavior of an ultrathin metal–polymer–metal hybrid system under complex transverse loading conditions has not been fully explored. Therefore, this study focuses on the experimental analysis of the mechanical properties and fracture mechanism of a steel–polymer/graphene–steel sandwich structure with an ultrathin total thickness (0.35 ± 0.01 mm). Through the evaluation of maximum load and displacement strain, the study clarifies the trade-off between increasing the shear strength of the system and the transition to brittle fracture mode, thereby guiding the application of this material in fields requiring high structural stability such as precision mechatronics and micro-components [16,23].

## 2. Materials and Methods

### 2.1. Materials and Sandwich Composite Processing

In this study, a metal–polymer–metal sandwich composite structure, reinforced with single-layer graphene, was designed and experimentally evaluated. The composite specimen consists of three distinct layers with a stacking sequence of steel/graphene-modified polymer/steel, yielding a total nominal thickness of 0.35 mm (experimentally verified as 0.35 ± 0.01 mm). Specifically, the configuration features top and bottom metallic face sheets (each 0.1 mm in thickness) to provide structural rigidity, while the intermediate core layer (0.15 mm in thickness) acts as a functional matrix comprising an alkyd-based coating uniformly dispersed with single-layer graphene. The detailed specifications of the constituent materials used to fabricate this sandwich structure are presented in the following subsections.

The material used in this study was a commercial austenitic stainless-steel foil, grade EN 1.4310 (equivalent to AISI 301). The material was supplied in a rolled format with a nominal thickness of 0.10 mm (Table 1). While specific yield strength and elongation of the thin foil were not independently tested via uniaxial tension due to equipment limitations for 0.1 mm films, the properties provided by the supplier indicate an ultimate tensile strength of 1500 to 1700 MPa. Identical batches with the same rolling direction were used for all test groups to ensure comparative validity.

The polymer matrix utilized as the intermediate layer in the sandwich composite was a commercial long-oil alkyd-based coating (JOTON JIMMY, light grey—LQ JOTON Joint Stock Company, Ho Chi Minh City, Vietnam). To achieve optimal rheological properties for the dispersion of single-layer graphene, the base polymer was diluted with a compatible aliphatic hydrocarbon solvent (commercially Jothinner-180—LQ JOTON Joint Stock Company, Ho Chi Minh City, Vietnam, commonly referred to as mineral turpentine) at a volumetric ratio of 3:1. The final chemical composition of the prepared mixture comprised 41.25 wt.% long-oil alkyd resin, 15.0 wt.% pigments (predominantly pure titanium dioxide with traces of carbon black), 7.5 wt.% mineral stabilizers, 11.25 wt.% aliphatic hydrocarbon solvent, and 25.0 wt.% of the added thinner. The fluid phase was modeled with a mixture density of 1020–1060 kg/m^3^, a dynamic viscosity of 0.05–0.07 Pa·s, and a surface tension of 25–27 mN/m. Furthermore, the physically based rendering (PBR) parameters use a light gray reflectance coefficient (Hex: #BFC1C2), a low surface roughness of 0.12, and a refractive index (IOR) of 1.52, indicating high gloss (Table 2).

**Figure 1:**
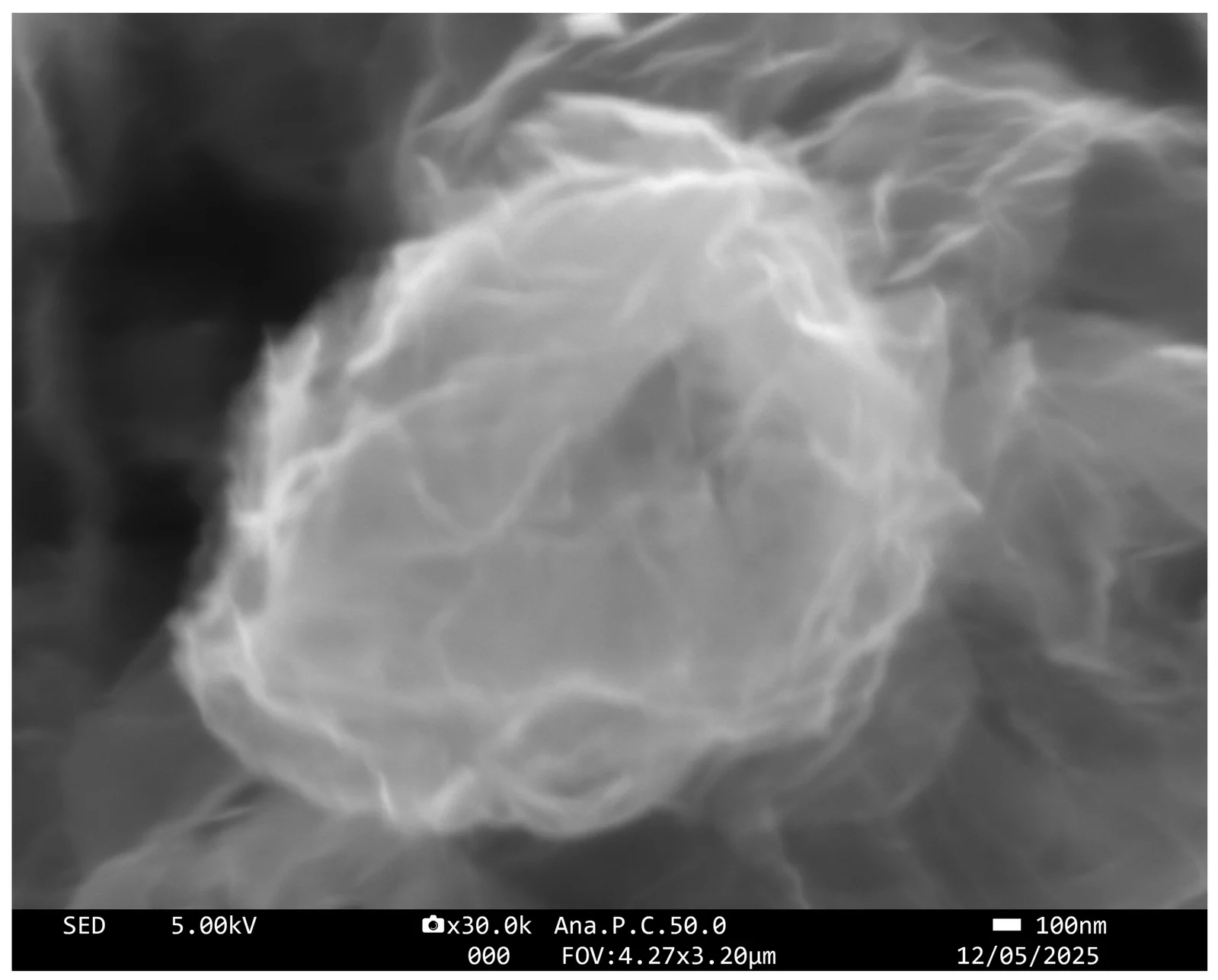
Image of single-layer graphene powder at 30,000× magnification.

The nanoparticulate filler utilized in this study was commercial single-layer graphene powder (Figure 1). According to the manufacturer’s technical specifications (as conventional SEM lacks the resolution to directly verify sub-nanometer thickness), the graphene flakes exhibit a lateral diameter ranging from 0.4 to 5 μm and a thickness between 0.6 and 1.2 nm. The material possesses a high specific surface area of 400–1000 m^2^/g, determined via the Brunauer–Emmett–Teller (BET) method. Additionally, the powder has a low apparent density of approximately 0.01 g/cm^3^ and an electrical resistivity of ≤0.30 Ω·cm. The intrinsic physical and electrical properties of the single-layer graphene powder, as specified by the supplier, are detailed in Table 3.

**Figure 2:**
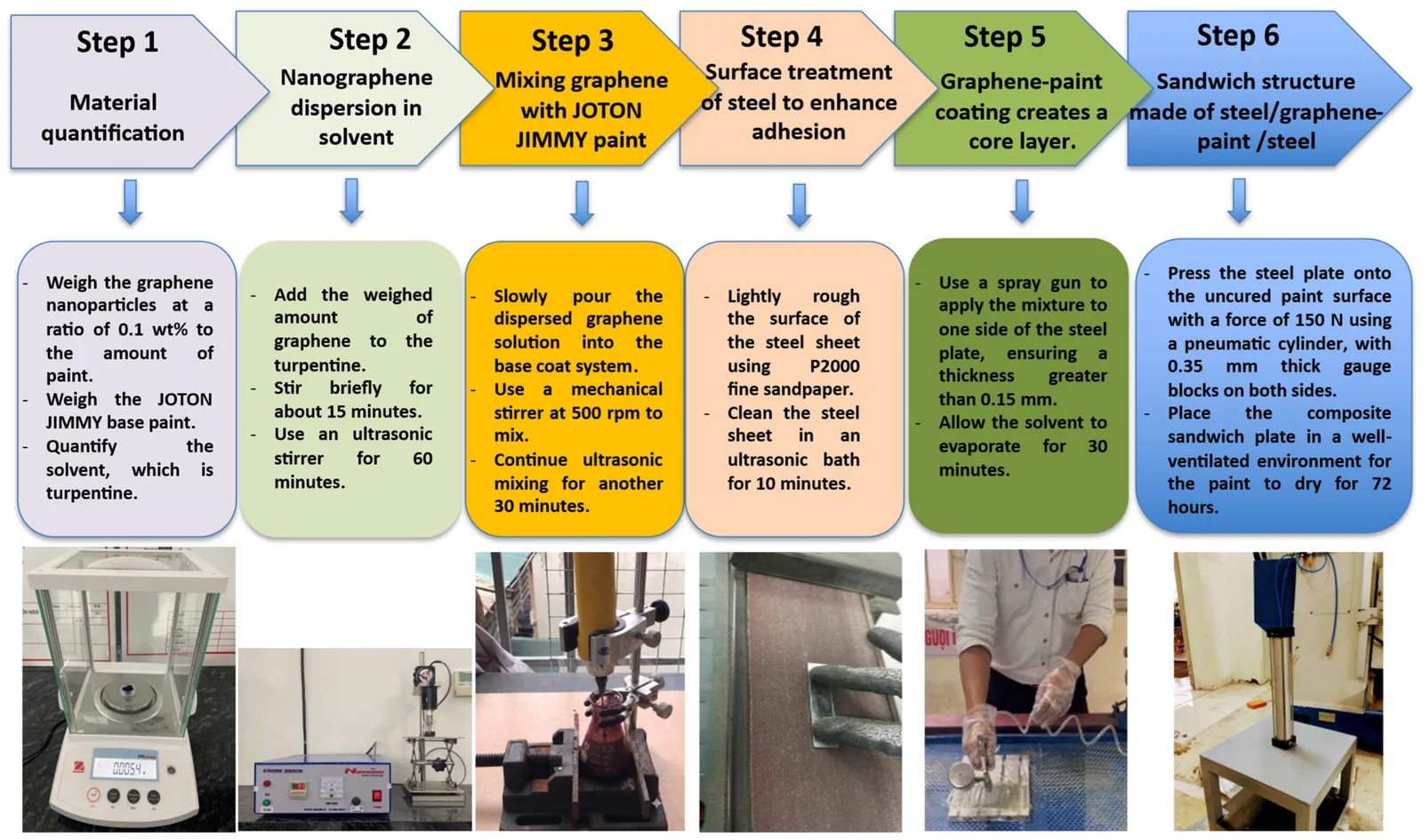
The process for fabricating a steel/graphene-reinforced polymer/steel sandwich composite.

The fabrication of steel/graphene-reinforced polymer/steel sandwich composite prototypes follows a step-by-step process, as shown in Figure 2.

During the synthesis of nanocomposite materials, especially when dispersing graphene into thermosetting resins such as alkyd-based polymer using processing equipment such as magnetic stirrers and ultrasonic vibrators, the optimal graphene content is usually maintained at a very low level, about 0.1 wt.% relative to the solid polymer resin fraction. The first reason stems from the morphological characteristics of this material. Furthermore, for highly viscous alkyd coatings that already contain 15 wt.% pigments and stabilizers, adding graphene beyond 0.1 wt.% causes severe mechanical mixing issues, restricts solvent evaporation, and promotes rapid re-agglomeration. Thanks to possessing an extremely large aspect ratio and specific surface area, graphene can quickly reach the percolation threshold to create an effective stress-transfer network at an extremely low load level [24,25]. When the content continues to increase beyond 0.1 wt.%, VdW forces between molecules will trigger agglomeration, causing graphene sheets to tend to stack on top of each other instead of being evenly dispersed in the resin [1,26]. These aggregates form stress-concentrating defects, degrading the mechanical properties such as tensile and compressive strength of the material [27]. Furthermore, the presence of chemical functional groups on the graphene surface when interacting with polymer chains will cause a sudden increase in the viscosity of the uncured solution system [28,29]. Excessively high viscosity creates a major barrier to mechanical mixing, making it difficult for air bubbles to escape and hindering tight bonding at the interface, thereby reducing the overall quality of the composite system [1,30]. In this study, the content of single-layer graphene mixed into the polymer was 0.1 wt.%. For thermosetting resins like JOTON JIMMY paint, as the solvent evaporates, the double bonds in the long-chain alkyd resin react with oxygen in the air to create a robust three-dimensional (3D) network. The 0.15 mm thin film of the alkyd–graphene mixture not only acts as an adhesive but also becomes a solid polymer-based composite material, adhering tightly to the micro-grooves on the surface of the EN 1.4310 thin steel sheet to transmit stress.

### 2.2. The Procedure for Performing the SPT Uses an Integrated Fixture on the INSTRON 3382A (Instron, Norwood, MA, USA) Universal Tensile and Compression Testing Machine

For thin sandwich composite materials (0.35 mm), the SPT method [31] was chosen in this study for the following reasons:

- For multilayer thin film samples with a total thickness of only 0.35 mm, standard three-point tensile or bending tests are difficult to perform. The clamping force of the tensile testing machine easily causes stress concentration, tearing the sample edges or causing crushing at the contact point, leading to large errors. SPT uses an annular clamping mechanism (upper and lower molds pressed together), which helps to keep the sample absolutely stable without damaging the structure.
- Unlike tensile tests, which only have uniaxial stress, SPT forces the material to withstand biaxial stress combined with shear force at the edge of the puncture. This most accurately reflects the actual load conditions of thin-film materials, such as impact from flying objects and local puncture pressure.
- Under local puncture pressure, the shear stress between the material layers (steel–polymer–steel) will reach its maximum level. SPT is the most visual method to test whether the graphene-reinforced alkyd core layer is capable of maintaining interfacial adhesion and resisting delamination.

**Figure 3:**
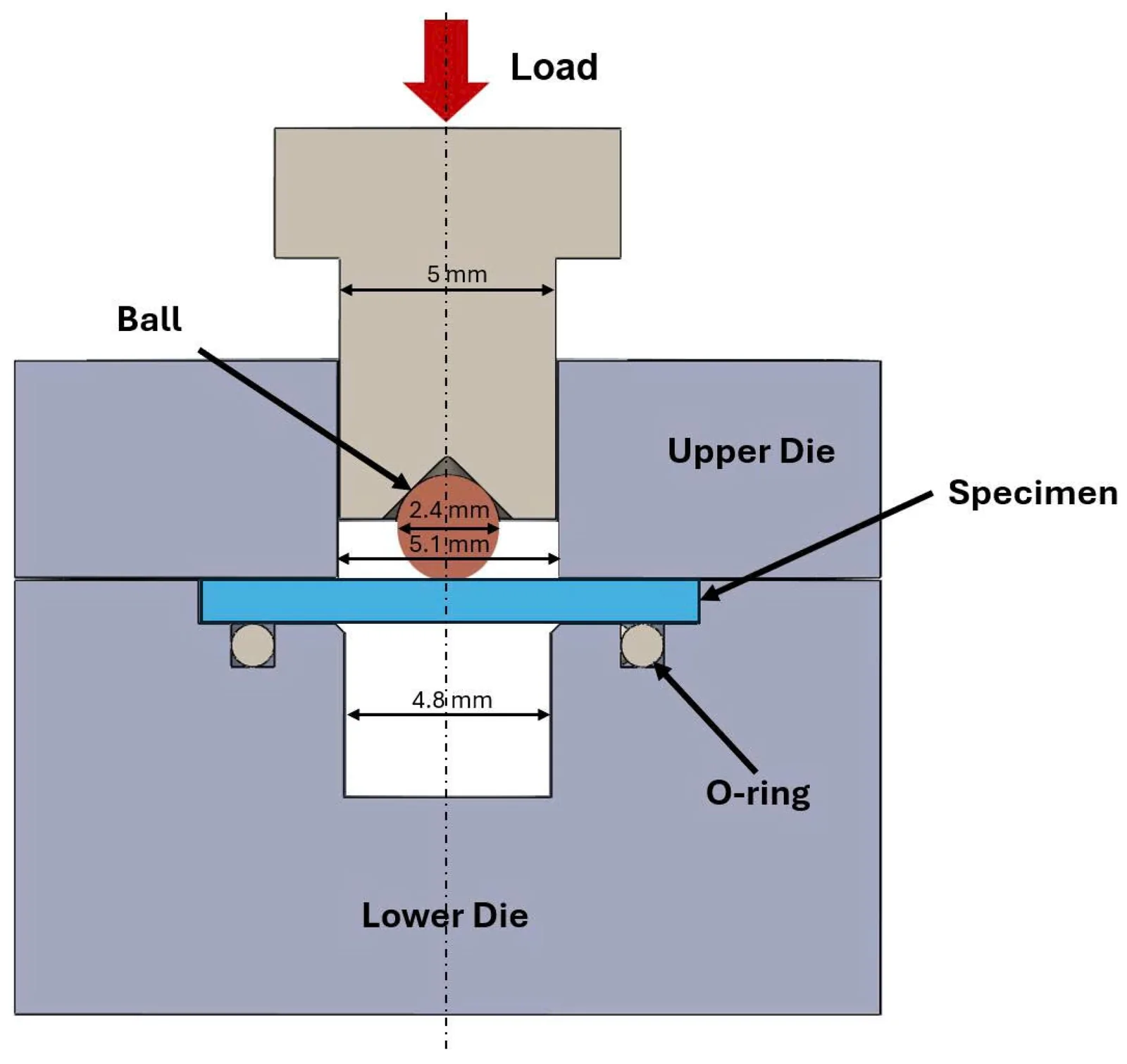
The structure of the SPT jig.

The SPT fixture has the following main geometric parameters: bottom die-hole diameter, 4.8 mm; punch ball diameter, 2.4 mm (Figure 3).

- With a punch ball diameter of 2.4 mm and a sample thickness of 0.35 mm, the ratio of penetration tip diameter to thickness is approximately 6.8. This is a very suitable ratio in deformation mechanics. If the ball is too small, it will cut through the metal layer immediately (punching) without enough time for force propagation. The ratio of 6.8 is large enough to force the sandwich structure to undergo bending and membrane stretching before being punctured, helping to collect accurate data on flexural stiffness and energy absorption capacity.
- The bottom die hole’s radius is 2.4 mm, and the punch ball radius is 1.2 mm. Therefore, the gap (space) from the edge of the punch ball to the bottom die edge is 1.2 mm, providing sufficient deformation space for the sandwich plate.
- This 1.2 mm gap is much larger than the material thickness (0.35 mm). This creates a “void space” that allows the material to be pulled downwards in a dome shape before tearing or brittle fracture. If the die hole is too small, the sample will be clamped tightly and undergo pure fracture. Thanks to the 4.8 mm hole diameter, a clear mechanical difference can be observed between the graphene sample that undergoes abrupt brittle fracture with shallow dome formation and the alkyd-core sample without graphene that tears slowly and has deep dome formation.

**Figure 4:**
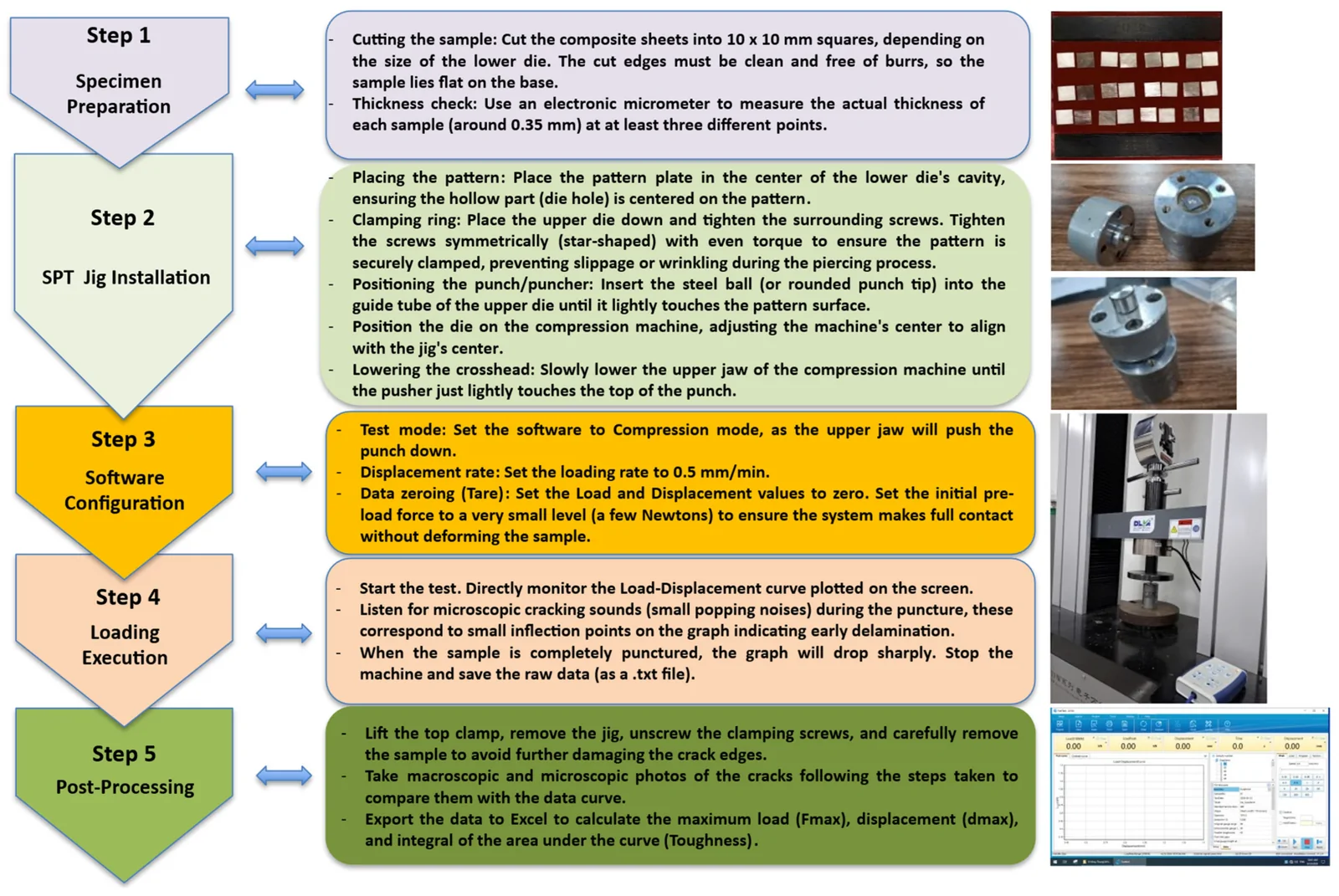
The process for performing the SPT method.

The experimental steps for evaluating the behavior and fracture morphology of steel/graphene-reinforced polymer/steel sandwich composite structures according to SPT are shown in Figure 4. For quasi-static testing on thin materials, the displacement rate is set very slowly to clearly observe the delamination process. In this study, the displacement rate was set at 0.5 mm/min.

To ensure the reliability of statistical data and eliminate random errors during fabrication, a total of 50 test specimens were evaluated. The specimens were divided into two groups: 25 control specimens without graphene and 25 specimens reinforced with graphene in the core layer. A two-sample *t*-test was conducted to verify the statistical significance of the differences in mechanical behavior. The results for maximum load, flexural stiffness, fracture strain, and energy absorption presented in this study are all statistical averages obtained from repeated experiments, ensuring representativeness of the mechanical behavior of the structure.

## 3. Results and Discussion

**Figure 5:**
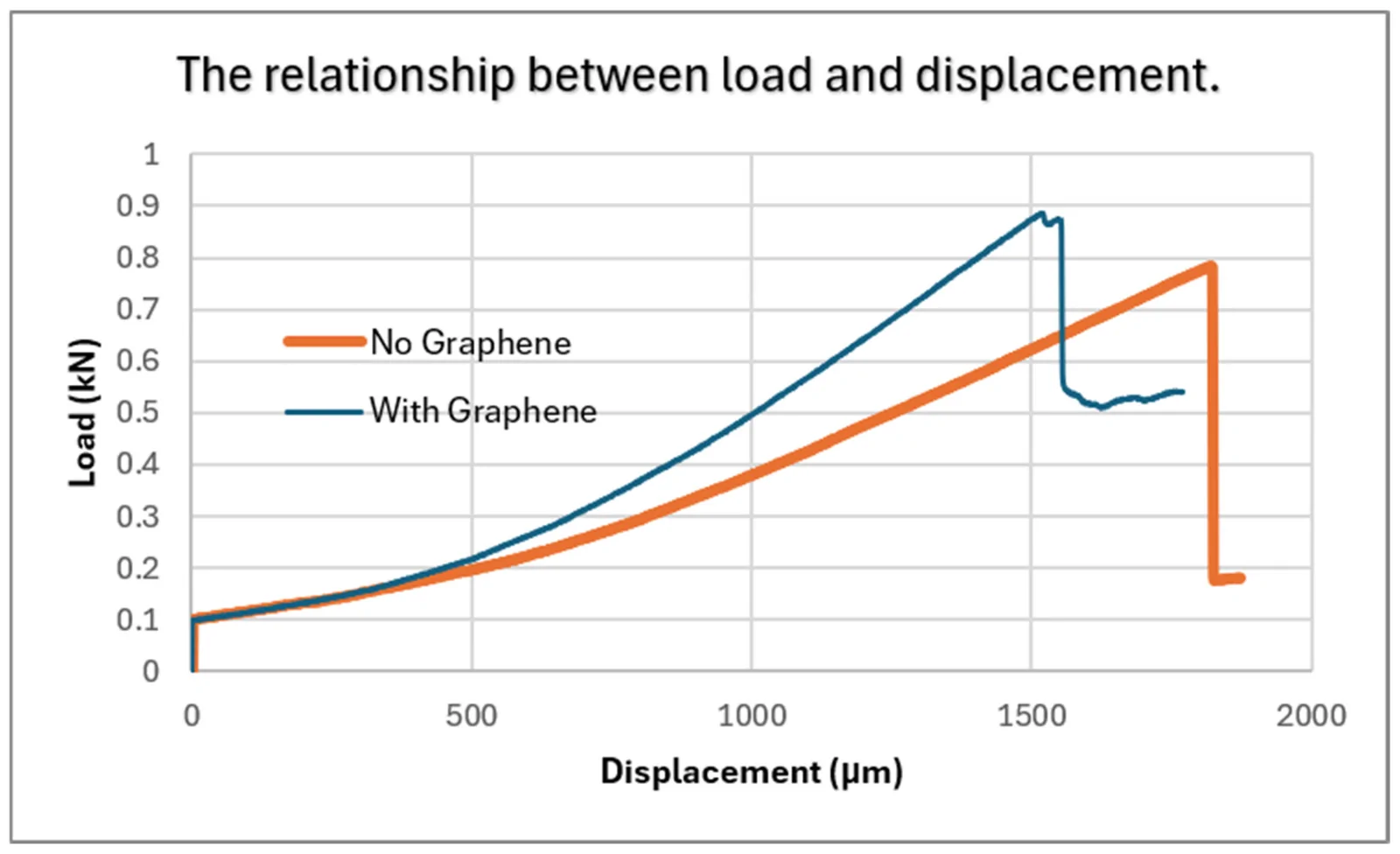
Comparative mechanical response of graphene-reinforced vs. unreinforced laminated sandwich composites under concentrated loading.

After performing statistical analysis and calculating the average values from 25 replicate tests for each sample group, the core mechanical parameters (peak load, deflection, and energy absorption) were established with confidence. With graphene reinforcement, the sandwich composite’s strength increased, but at the same time, the material’s initial plasticity decreased. Statistical mechanical properties of the sandwich composite structures obtained from SPTs (*n* = 25 per group) are shown in Table 4. The results include the mean, standard deviation (SD), and coefficient of variation (CoV). The exceptionally low CoV values (under 10%) macroscopically indicate that the graphene was uniformly dispersed within the alkyd matrix, avoiding severe stress-concentrating agglomerations. To clarify the mechanical nature and the transformation in the fracture mechanism, Figure 5 presents two typical load–displacement curves, representing the characteristic experimental behavior of the non-graphene and with-graphene sample groups under small puncture loads.

### 3.1. Analysis of Mechanical Mechanisms and Fractures in the SPT Method

#### 3.1.1. Peak Load Increase Mechanism

The 12.6% increase in peak load (from 0.79 kN to approximately 0.89 kN) is primarily due to the efficient stress transfer capabilities of the graphene network.

- Graphene has an extremely high elastic modulus and tensile strength. When dispersed in a 0.15 mm thick alkyd core, the graphene sheets are hypothesized to act as the primary load-bearing framework.
- Under the action of the punch ball, stress from the local contact region is transferred through the softer polymer matrix to the stiffer graphene networks via interfacial bonding. This increases the overall stiffness of the core layer, preventing premature local settlement and allowing the structure to withstand greater compressive forces before reaching the fracture critical point.

#### 3.1.2. Displacement Reduction Mechanism

Although increasing strength, the addition of graphene reduced the critical strain travel by 16.7% (from 1800 µm to 1500 µm), clearly demonstrating the partial embrittlement of the structure. We hypothesize that graphene sheets have a large aspect ratio, creating spatial obstacles that lock and significantly reduce the plastic sliding ability of the polymer chains in the alkyd matrix.

- Because the core layer becomes stiffer, the structure loses some of its ability to absorb large plastic deformations before failure. The material reacts more aggressively, causing fracture initiation to occur earlier and at a lower deflection level compared to the sample without graphene.

#### 3.1.3. Bending and Membrane Stress Distribution

A key characteristic of the SPT method on ultrathin films is the rapid transition from the bending to the tensile state of the film.

- In the initial stage, graphene reinforcement increases the local flexural modulus of the core layer, helping to disperse the force from the punch ball over a wider area instead of concentrating it at a single tear point.
- As the deflection increases, the composite core layer is strongly stretched. The graphene network helps distribute the film stress more evenly across the 0.15 mm plane of the core, delaying early necking at the bottom steel layer surface, allowing the structure to maintain its integrity at higher load levels.

#### 3.1.4. Transverse Shear Capacity

With a die diameter of 4.8 mm and a punch tip diameter of 2.4 mm, the clearance area is subjected to severe shear stress.

- Conventional polymer cores are very weak under shear stress. However, the interlocking of graphene sheets is hypothesized to create a mechanical interlocking mechanism. Graphene may restrict the shear sliding of the polymer planes against each other.
- The improved shear resistance of the core prevents the punch ball from easily penetrating the system at a right angle; the punch force is converted into tensile energy throughout the system, thereby increasing the maximum load limit.

#### 3.1.5. Failure Mode and Plastic Deformation

The most noticeable change lies in the macroscopic fracture pattern observed on the graph.

- The non-graphene sample exhibits ductile tearing with large plastic deformation. The core acts as a cushion, allowing significant macroscopic deflection.
- The with-graphene sample exhibits an abrupt brittle shear plug failure. The sample has a shorter plastic deformation zone, abruptly failing when the load limit is reached without significant macroscopic necking.

### 3.2. Energy Dissipation Mechanism

To quantitatively assess the comparative energy absorption of the structure, it is necessary to calculate the area under the load–displacement curve from the start of loading until the maximum load (Fmax) is reached. Because the load–displacement curves are highly nonlinear, the integrated energy index was calculated by numerically integrating the raw experimental data using the trapezoidal rule.

Although graphene blending helps the core matrix withstand 12.6% higher maximum load (from 0.79 kN to 0.89 kN), it results in a slight reduction in the integrated energy index of the system by 6.1% (from 0.711 J to 0.6675 J).

The relatively higher 0.711 J value of the non-graphene sample suggests better plasticity and stress dispersion. Energy is slowly absorbed by the material to create greater macroscopic deflection (up to 1.8 mm) and maintain material bridges at the crack. Conversely, the slightly reduced energy level of 0.6675 J of the graphene sample, coupled with its shorter critical strain travel (1.5 mm), indicates a tendency toward embrittlement. This suggests that the structure is more rigidly locked, restricting its ability to dissipate energy through extensive plastic deformation and instead accumulating it as elastic potential energy, leading to an earlier and more abrupt fracture.

### 3.3. Analysis of the Images of Experimental Specimens

**Figure 6:**
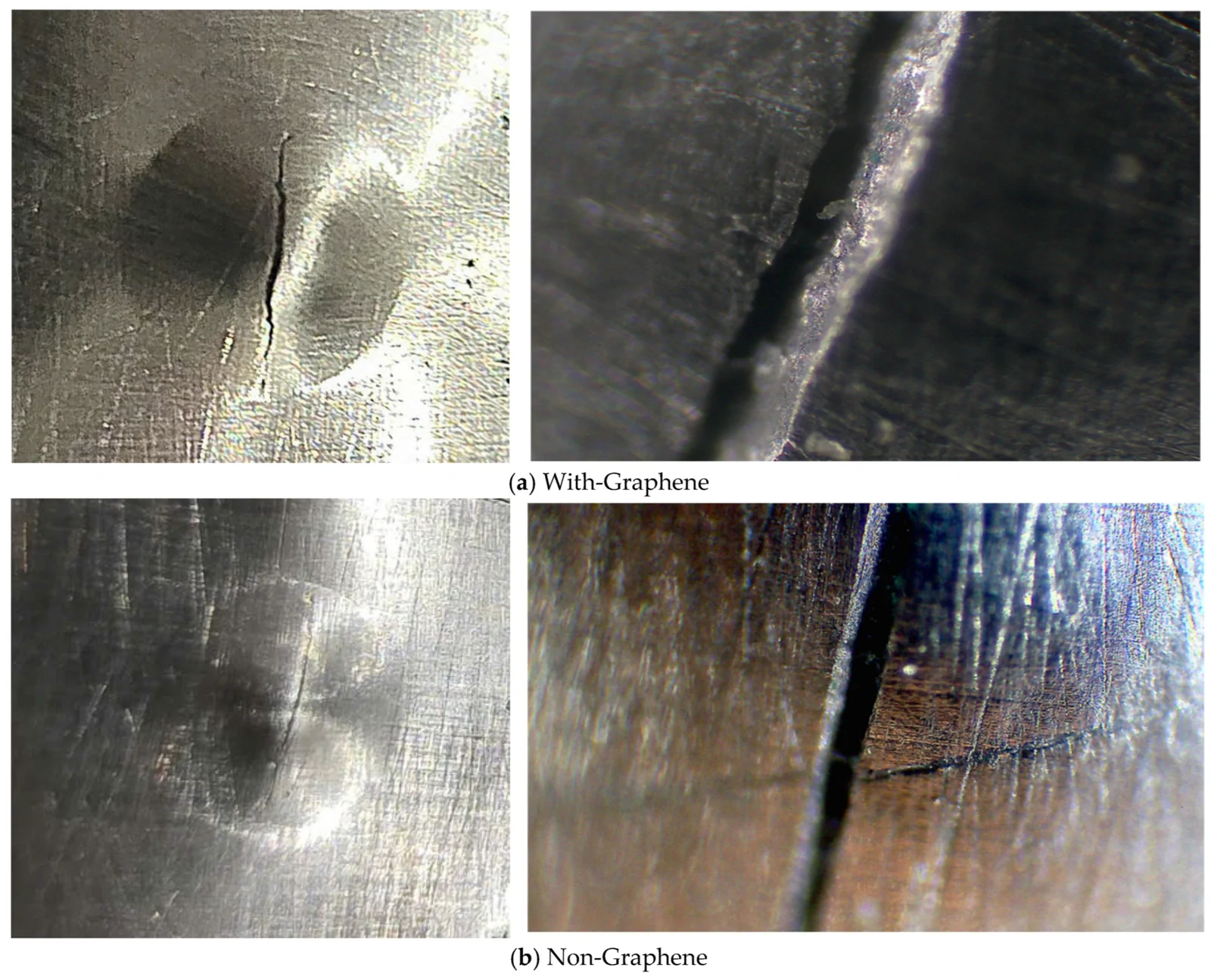
The sample image was destroyed after the experiment.

Based on the quantitative analyses of fracture mechanics and load–displacement diagrams in the previous section, it is possible to compare and explain the physical phenomena observed on the fracture surface of the samples through experimental images (Figure 6).

#### 3.3.1. Dome Formation Assessment

Comparison of two overall sample images after puncture (with graphene and without graphene):

- Non-Graphene Sample: The dome-shaped indentation expands and spreads outwards around the puncture area. The steel surface around the crack has a large curvature, indicating that the entire structure underwent significant bending and membrane stretching before tearing. This perfectly matches the long puncture stroke (1.8 mm) on the graph.

The fact that the crack did not completely separate and block light, even though the ball puncture stroke was very deep (1.8 mm), is evidence of plastic tearing and fracture delay. Material Bridging Hypothesis: The original unfilled alkyd core is softer, allowing the two steel surfaces to bend and slide against each other. As the puncture penetrates downwards, the steel layer on the underside (tension side) begins to tear, but the adhesive layer and possibly part of the steel layer on the top side are still plastically stretched to “hold” the two edges of the crack together. Residual Structural Integrity: Although the graph has passed the peak load (0.79 kN) and the load has dropped, the actual structure has not completely broken into two parts. The material at the tear still maintains a fragile bond. This indicates that the material has a very good ability to absorb excess energy through macroscopic plastic deformation before complete fracture.

- With-Graphene Sample: The indentation is localized and much narrower. The area of material around the crack is less prone to sagging, maintaining relative flatness compared to the sample without graphene. The puncture is “abrupt” at the point of maximum compressive force, reflecting high stiffness and a short penetration stroke (1.5 mm). Indications of Brittle-Like Failure: Upon reaching the load limit (0.89 kN), the graphene–alkyd network became too rigid to stretch further. The sudden release of stress suggests a rapid, localized structural failure rather than gradual tearing. The image shows that the two edges of the crack have physically separated. The previously accumulated elastic compressive force springs back, pushing the two torn metal edges apart, creating a sufficiently large and visible gap. The structure at the puncture point has completely lost its load-bearing capacity.

#### 3.3.2. Macroscopic Crack Morphology Analysis

When observing magnified optical images of the crack surface, the differences in tearing mechanisms become apparent:

Non-Graphene Sample (Ductile Tearing Tendency):

- Crack opening: The crack is very wide and tends to split open.
- Crack edge: The crack edge is relatively blunt, with the metal ridges showing signs of being pulled thin and tapering downwards before complete fracture.
- Surface deformation traces: Around the crack, macroscopic wrinkling and localized sagging parallel to the crack edge can be observed. This is evidence of plastic flow in the 0.1 mm steel shell. Because the underlying alkyd resin is soft, it acts as a “cushion,” allowing the two steel surfaces to slide macroscopically and undergo maximum plastic elongation before the tensile stress exceeds the fracture limit.

With-Graphene Sample (Brittle Shear Tendency):

- Crack opening: Narrower, more localized cracks.
- Crack edge: The tear surface is sharp, jagged, and serrated. Macroscopically, there is significantly less yielding or sagging at the steel edge compared to the unfilled sample.
- Top-down optical observation: While high-resolution cross-sectional fractography is required to observe the exact microcracking networks and layer delamination, the top-down optical image shows a distinct lack of widespread plastic yielding. We hypothesize that the graphene-infused alkyd core is very rigid, strongly restricting the mobility of the structural layers. When the indenter penetrates downwards, the shear stress is maximally concentrated at the contact edge. Instead of plastic stretching, the structure resists until it reaches peak load (0.89 kN), after which it undergoes a rapid failure (suggestive of a localized shear-plug mechanism). The macroscopic fracture appears to occur so quickly that the material does not have time to plastically deform to dissipate the energy, creating sharper and steeper crack edges.

## 4. Conclusions

Through the small punch test (SPT) method, a study was conducted to evaluate the mechanical properties and macroscopic fracture behavior of a thin steel/graphene-reinforced polymer/steel sandwich structure (with a measured total thickness of 0.35 ± 0.01 mm).

Dispersing the graphene network into the alkyd core layer significantly improved the structural flexural stiffness and increased the maximum puncture load by 12.6%, suggesting efficient stress transfer from the polymer matrix to the 2D material framework under localized loading. However, while well-dispersed graphene is frequently reported to increase fracture toughness via microcrack bridging and crack deflection in unfilled polymers, our macroscopic results indicate a different dominant mechanism in this highly filled alkyd matrix.

We hypothesize that the addition of graphene into this densely packed system exerts a severe spatial locking effect, strongly restricting the mobility of the alkyd polymer chains. While this constraint increases peak load capacity, it results in a 16.7% reduction in the critical strain travel (from 1800 µm to 1500 µm) and a slight reduction in approximated energy absorption. This indicates a clear tendency toward material embrittlement during the initial bending and stretching phases, leading to an earlier and more abrupt fracture initiation rather than ductile yielding.

To fully exploit the potential of thin sandwich composite systems, future research must address current experimental limitations. High-resolution cross-sectional fractography (such as FESEM, TEM, or AFM) combined with elemental mapping is required to directly observe the nanoscale interfacial mechanics and definitively verify the hypothesized chain-locking and micro-delamination phenomena. Additionally, independent uniaxial tensile testing of the ultrathin steel foils is necessary to isolate the core’s exact contribution. Synchronous evaluations under dynamic impact, cyclic fatigue, and long-term creep loads will also be conducted. These fundamental optimizations will create a solid foundation for bringing this composite system into practical applications, such as precision mechatronics and advanced protective shells.

## Figures and Tables

**Table 1:** Properties of the as-received EN 1.4310 stainless steel foil.

Parameter	Value	Unit
Material Grade	EN 1.4310 (AISI 301)	-
Nominal Thickness	0.1	mm
Tensile Strength	1500–1700	MPa

**Table 2:** Physicochemical, rheological, and optical parameters of the prepared alkyd polymer matrix.

Parameter	Value	Unit
Base-to-Thinner Ratio	3:1	-
Long-Oil Alkyd Resin	41.25	wt.%
TiO_2_ & Carbon Black trace	15.0	wt.%
Mineral Stabilizers	7.5	wt.%
Aliphatic Hydrocarbons	11.25	wt.%
Added Thinner (Jothinner-180)	25.0	wt.%
Density	1020–1060	kg/m^3^
Dynamic Viscosity	0.05–0.07	Pa·s
Surface Tension	25–27	mN/m
Base Color (Albedo)	Light Grey (#BFC1C2)	-
Roughness	0.12 (High gloss)	-
Index of Refraction (IOR)	1.52	-

**Table 3:** Physicochemical properties of the single-layer graphene powder.

Property	Value	Unit
Morphology	Single-layer flakes	-
Flake Diameter	0.4–5	µm
Thickness	0.6–1.2	nm
BET Surface Area	400–1000	m^2^/g
Electrical Resistivity	≤0.30	Ω·cm
Apparent Density	~0.01	g/cm^3^

**Table 4:** Statistical mechanical properties of the sandwich composite structures obtained from SPTs (*n* = 25 per group).

Group	Peak Load (kN)		Displacement (mm)	
	Mean ± SD	CoV	Mean ± SD	CoV
Non-Graphene	0.79 ± 0.03	3.8%	1.80 ± 0.08	4.4%
With-Graphene	0.89 ± 0.04	4.5%	1.50 ± 0.09	6.0%
*p*-value	<0.001		<0.001	

## Data Availability

Data are contained and presented within the article.

## Abbreviations

The following abbreviations are used in this manuscript:

SPT	small test punch
CNTs	carbon nanotubes
VdW	van der Waals

## References

[1] Lee S.J., Yoon S.J., Jeon I.-Y. (2022). Graphene/Polymer Nanocomposites: Preparation, Mechanical Properties, and Application. Polymers.

[2] Ibrahim A., Klopocinska A., Horvat K., Hamid Z.A. (2021). Graphene-Based Nanocomposites: Synthesis, Mechanical Properties, and Characterizations. Polymers.

[3] Tarannum F., Muthaiah R., Annam R.S., Gu T. (2020). Effect of Alignment on Enhancement of Thermal Conductivity of Polyethylene–Graphene Nanocomposites. Nanomaterials.

[4] Dehnou K.H., Norouzi G.S., Majidipour M. (2023). A review: Studying the effect of graphene nanoparticles on mechanical, physical and thermal properties of polylactic acid polymer. RSC Adv..

[5] Kausar A., Ahmad I., Eisa M.H., Maaza M. (2023). Graphene Nanocomposites in Space Sector—Fundamentals and Advancements. C.

[6] Díez-Pascual A.M., Rahdar A. (2022). Graphene-Based Polymer Composites for Flexible Electronic Applications. Micromachines.

[7] Thompson M.S., Agarwal S., Gupta R.K. (2011). Barrier Properties of Graphene-Based Polymer Nanocomposites. Mt. Undergrad. Res. Rev..

[8] Atif R., Shyha I., Inam F. (2016). Mechanical, Thermal, and Electrical Properties of Graphene-Epoxy Nanocomposites—A Review. Polymers.

[9] Zhang X., Zheng J., Ma Z. (2019). Graphene-polymer Composites for Enhancing the Mechanical Properties. IOP Conf. Ser. Mater. Sci. Eng..

[10] Liu L., Xu C., Yang Y., Fu C., Ma F., Zeng Z., Wang G. (2025). Graphene-based polymer composites in thermal management: Materials, structures and applications. Mater. Horiz..

[11] Mazzuca P., Firmo J.P., Correia J.R., Rosa I.C. (2025). Fire behaviour of vacuum-infused glass fibre reinforced polymer sandwich panels: The role of polyurethane and polyethylene terephthalate foam cores and panel architecture. Eng. Struct..

[12] Kostagiannakopoulou C., Loutas T.H., Sotiriadis G., Markou A., Kostopoulos V. (2015). On the interlaminar fracture toughness of carbon fiber composites enhanced with graphene nano-species. Compos. Sci. Technol..

[13] Liu Y., Zou A., Wang G.-D., Han C., Blackie E. (2022). Enhancing interlaminar fracture toughness of cfrp laminates with hybrid carbon nanotube/graphene oxide fillers. Diam. Relat. Mater..

[14] Young R.J., Kinloch I.A., Gong L., Novoselov K.S. (2012). The mechanics of graphene nanocomposites: A review. Compos. Sci. Technol..

[15] Papageorgiou D.G., Kinloch I.A., Young R.J. (2017). Mechanical properties of graphene and graphene-based nanocomposites. Prog. Mater. Sci..

[16] Potts J.R., Dreyer D.R., Bielawski C.W., Ruoff R.S. (2011). Graphene-based polymer nanocomposites. Polymer.

[17] Chandrasekaran S., Sato N., Tölle F., Mülhaupt R., Fiedler B., Schulte K. (2014). Fracture toughness and failure mechanism of graphene based epoxy composites. Compos. Sci. Technol..

[18] Khalid M.Y., Kamal A., Otabil A., Mamoun O., Liao K. (2023). Graphene/epoxy nanocomposites for improved fracture toughness: A focused review on toughening mechanism. Chem. Eng. J. Adv..

[19] Bortz D.R., Heras E.G., Martin-Gullon I. (2012). Impressive fatigue life and fracture toughness improvements in graphene oxide/epoxy composites. Macromolecules.

[20] Ji X.-Y., Cao Y.-P., Feng X.-Q. (2010). Micromechanics prediction of the effective elastic moduli of graphene sheet-reinforced polymer nanocomposites. Model. Simul. Mater. Sci. Eng..

[21] Young R.J., Liu M., Kinloch I.A., Li S., Zhao X., Vallés C., Papageorgiou D.G. (2018). The mechanics of reinforcement of polymers by graphene nanoplatelets. Compos. Sci. Technol..

[22] Guo G., Zhu Y. (2015). Cohesive-shear-lag modeling of interfacial stress transfer between a monolayer graphene and a polymer substrate. J. Appl. Mech..

[23] Chen W., Yang T., Dong L., Elmasry A., Song J., Deng N., Elmarakbi A., Liu T., Lv H.B., Fu Y.Q. (2020). Advances in graphene reinforced metal matrix nanocomposites: Mechanisms, processing, modelling, properties and applications. Nanotechnol. Precis. Eng..

[24] Istrate O.M., Paton K.R., Khan U., O’Neill A., Bell A.P., Coleman J.N. (2014). Reinforcement in melt-processed polymer–graphene composites at extremely low graphene loading level. Carbon.

[25] Ramanathan T., Abdala A.A., Stankovich S., Dikin D.A., Herrera-Alonso M., Piner R.D., Adamson D.H., Schniepp H.C., Chen X., Ruoff R.S. (2008). Functionalized graphene sheets for polymer nanocomposites. Nat. Nanotechnol..

[26] Sun X., Huang C., Wang L., Liang L., Cheng Y., Fei W., Li Y. (2021). Recent progress in graphene/polymer nanocomposites. Adv. Mater..

[27] Akter M., Ozdemir H., Bilisik K. (2024). Epoxy/graphene nanoplatelet (GNP) nanocomposites: An experimental study on tensile, compressive, and thermal properties. Polymers.

[28] Rabchinskii M.K., Shiyanova K.A., Brzhezinskaya M., Gudkov M.V., Saveliev S.D., Stolyarova D.Y., Torkunov M.K., Chumakov R.G., Vdovichenko A.Y., Cherviakova P.D. (2024). Chemistry of reduced graphene oxide: Implications for the electrophysical properties of segregated graphene–polymer composites. Nanomaterials.

[29] Xu Z., Gao C. (2010). In situ polymerization approach to graphene-reinforced nylon-6 composites. Macromolecules.

[30] da Luz F.S., Garcia Filho F.d.C., del-Río M.T.G., Nascimento L.F.C., Pinheiro W.A., Monteiro S.N. (2020). Graphene-incorporated natural fiber polymer composites: A first overview. Polymers.

[31] (2026). Standard Test Method for Small Punch Testing of Metallic Materials.

